# Lessons Learned From the Implementation of a Clinic-Focused HPV Vaccination Initiative

**DOI:** 10.1177/21501319261428038

**Published:** 2026-03-18

**Authors:** Idara N. Akpan, Grace Maynard, Rachel J. Meadows, Kimberly G. Fulda, Divya A. Patel, Aaron W. Gehr, Yan Lu, Sarah Matches, Anna Espinoza, Erika L. Thompson

**Affiliations:** 1 University of North Texas Health Science Center, Fort Worth, TX, USA; 2 JPS Health Network, Fort Worth, TX, USA; 3 UTHealth Houston School of Public Health in Austin, TX, USA; 4 The University of Texas at San Antonio, TX, USA

**Keywords:** human papillomavirus, HPV vaccination, quality improvement initiative, healthcare providers, health communication, Plan-Do-Study-Act model

## Abstract

**Introduction:**

Strengthening provider recommendation is an effective strategy in increasing the human papillomavirus (HPV) vaccine uptake. A quality improvement (QI) initiative called “Communicating about HPV to Adults and Teens” (HPV CHAT) was initiated to support providers with communication tools to discuss HPV vaccination with parents and patients. We described lessons learned for implementation of a clinic-based provider training for HPV vaccination.

**Methods:**

HPV CHAT was implemented in safety-net hospital clinics, practice-based research clinics, and federally qualified health centers between September 2021 and August 2023. Guided by the Plan-Do-Study-Act (PDSA) model, we retrospectively evaluated process improvements across 2 implementation cycles.

**Results:**

Overall, 318 providers participated across the 2 cycles. Key activities in the first cycle included identification of clinic needs, development of asynchronous virtual training, advisory board feedback, recruitment communications via practice and clinic managers, and provider participation monitoring. Barriers included slow response to participation (58.3% response rate) and high turnover rate in some clinics. Results from the first cycle informed activities implemented in the second cycle. In the second cycle, participation rate was 60.6% and varied across settings due to strength of clinic collaborations and prior knowledge of the implementation process.

**Conclusions:**

The success of HPV CHAT underscores the need to proactively tailor interventions for clinical settings.

## Introduction

Over 80% of sexually active adults will be infected with human papillomavirus (HPV) during their lifetime.^1^ Vaccination is a primary preventive strategy against HPV infection, which is the leading cause of anogenital and oropharyngeal cancers.^2-4^ HPV vaccination is recommended for children and adolescents ages 11 to 12 years (and as early as age 9), with catch-up vaccination up until age 26.^5^ Among 27-to-45-year-olds, the HPV vaccine is considered based on shared clinical decision-making between a healthcare provider and patient.^5^

Despite evidence of HPV vaccine safety and efficacy,^6^ vaccine hesitancy toward HPV vaccination remains among parents and patients.^7-12^ Consequently, HPV vaccination rates among U.S. adolescents are suboptimal with only 61.4% of 13 to 17-year-olds up to date in 2023.^13-15^ Insufficient information about the HPV vaccine, including the rationale for the vaccine and possible side effects are some of the reasons for parental refusal or hesitation.^8,9,16^ High-quality provider recommendation can improve uptake and completion of the HPV vaccine series.^17-22^ Nonetheless, provider recommendations are often weak and insufficient.^23^ Communication strategies, such as presumptive recommendation that assumes parents are ready to vaccinate and motivational interviewing, can strengthen the effectiveness of provider recommendations.^24^ Equipping healthcare providers with evidence-based strategies is crucial for navigating quality conversations about HPV vaccination, especially among vaccine-hesitant parents or patients.

Recognizing the critical need to address low uptake of HPV vaccination rates, we developed a brief asynchronous educational training called, “Communicating about HPV to Adults and Teens” (HPV CHAT). The HPV CHAT methodology and immediate outcomes have been published elsewhere.^25,26^ The idea for HPV CHAT evolved through collaborative discussions among subject-matter experts, including healthcare professionals, researchers, and community advocates who identified the urgency to improve communication and recommendations for HPV vaccinations. Briefly, the core function of the training was to teach effective communication strategies to empower clinicians, nurses, and clinical staff to confidently recommend HPV vaccination and address patient concerns.

Provider engagement in quality improvement initiatives (QI) is critical to successful implementation of evidence-based practices and potential effectiveness of provider-focused interventions.^27^ Nevertheless, barriers to provider involvement are common. For example, lack of supportive leadership that appropriately incentivizes and provides dedicated time for provider involvement in quality improvement initiatives can be a major barrier to provider participation.^28^ Additionally, interventions must be well-aligned with health system priorities and needs, such as health condition areas, cost and time required, project timelines, and type of interventions.^29^ Lack of goal alignment and preferred implementation strategies between researchers and health system partners can contribute to suboptimal provider motivation to participate in QI initiatives.^30^

Utilizing established frameworks like Plan-Do-Study-Act (PDSA) provides a structured approach to guiding and evaluating implementation strategies in healthcare settings, thereby strengthening evidence-based practices.^31-33^ Using the PDSA framework not only provides a systematic analysis of HPV CHAT rollout in clinical settings but also serves as a valuable case study for refining broader implementation strategies of communication tools in healthcare settings. Ultimately, this approach contributes to the broader goal of enhancing the adoption of evidence-based interventions to facilitate strong HPV vaccination recommendations.

The program implementation did not use PDSA methodology in real-time. Our paper uses the tenets of PDSA to retrospectively analyze and describe lessons learned during HPV CHAT implementation. We described lessons learned during the integration of HPV CHAT as a QI initiative to aid buy-in and engagement among healthcare providers with the aim to increase HPV vaccine uptake. Using PDSA principles, we illustrate the iterative approach initiated during implementation to facilitate adoption, feasibility, and integration of HPV CHAT within routine clinical activities in 2 iterations of program delivery. This paper aims to fill critical gaps in understanding effective strategies and overcoming challenges in HPV vaccination.

## Methods

### Program

HPV CHAT was launched across community and county clinical teams. The participating sites included safety-net hospital clinics, practice-based research clinics, health department immunization clinics, and Federal Qualified Health Centers (FQHCs). Key partners included the North Texas Primary Care Practice-Based Research Network (NorTex), John Peter Smith Health Network (JPS), and the Immunization Collaboration of Tarrant County (ICTC).

Following the implementation of HPV CHAT, participants reported positive impacts on both confidence and knowledge levels. These improvements were consistently observed across all clinical roles, albeit to varying degrees.^25^ In summary, this QI initiative successfully enhanced communication skills among diverse clinical roles. In addition, the provider feedback intervention, while showing no substantial impact on overall HPV vaccination rates in a subset of the QI clinics, results revealed modest increases in HPV vaccination initiation among individuals aged 9 to 12 and 13 to 17 years.^26^

Given the short-term outcomes from HPV CHAT with modest long-term impact, there is need to further evaluate what implementation strategies may have hindered or supported adoption of the QI training. Furthermore, to address the scarcity of studies documenting implementation strategies in clinical settings, there is a need for research that explicitly delves into the implementation process of interventions such as the HPV CHAT training.^34^

### Implementation

The HPV CHAT initiative was implemented in 2021 (Cycles 1 and 2). There were 18 clinics (Cycle 1) and 7 clinics (Cycle 2) that participated. The clinic liaisons at each network/clinic were instrumental in recruitment, establishing direct communication channels, building trust, and seamlessly integrating HPV CHAT into clinic workflows.

In Cycle 1, there were 7 safety-net hospital clinics, 2 practice-based research clinics, and 9 health department immunization clinics. During the recruitment phase of the HPV CHAT initiative, HPV CHAT was piloted at practice-based research clinics in April 2022. Following this successful pilot, the initiative was launched in the remaining safety-net hospital and health department immunization clinics. For Cycle 1, our goals were to train clinic teams, immunization staff, and volunteers on HPV vaccine communication skills, evaluate knowledge on HPV vaccine guidelines and self-efficacy to communicate with parents and patients about HPV vaccination. Additionally, we aimed to distribute HPV vaccination materials to parents and patients.

Upon securing funds for Year 2, HPV CHAT collaborated with FQHCs for the deployment of HPV CHAT. Sustaining collaboration with a safety-net hospital system, we targeted 4 clinics with low HPV vaccination rates. Year 2 included a soft launch at safety-net hospital clinics in April 2023 and deployment to FQHCs in May 2023. Similar to Cycle 1, we trained clinic teams on HPV vaccine communication skills and evaluated knowledge on HPV vaccine guidelines and self-efficacy to communicate with parents and patients about HPV vaccination. See Table 1 for detailed description of clinics.

**Table 1.** HPV CHAT Participating Clinics, 2021 to 2023.

Participating clinics	Cycle 1: 2021-2022	Cycle 2: 2022-2023
Safety-net hospital clinics	7 clinics • 1 Pediatric • 2 School-Based • 4 Community-based	4 clinics • 1 Women’s clinic • 1 Family Medicine • 2 Community-based
Practice-based research clinics	2 clinics • 1 Pediatric • 1 Family Medicine	3 clinics • 3 FQHCs
Health department immunization clinics	9 county immunization clinics	

Abbreviation: FQHCs, Federal Qualified Health Centers.

In this project, we employed various implementation strategies, including meals and incentives, utilizing clinic champions for ongoing communication, sending reminder emails, supplying patient education materials and training summaries, and offering monthly clinic-level audit-and-feedback data reports on HPV vaccination rates distributed through practice managers.^25^

This study was reviewed by the North Texas Regional Institutional Review Board and deemed not to be human subjects research. This manuscript was prepared in accordance with the SQUIRE reporting guidelines for quality improvement in health care.^35^

## Results

To describe lessons learned at each implementation cycle, we retrospectively applied the PDSA principles to identify challenges or gaps and possible solutions (*Plan*), implement the planned strategies (*Do*), analyze and review outcomes (*Study*), and use the information gathered to make modifications and guide subsequent steps (*Act*). Additionally, we used process notes to inform the evaluation, while also having iterative discussions with the program team through the project period.

### Cycle 1 (September 2021-August 2022)

The *Plan* phase included identifying the gaps in HPV vaccine recommendation during healthcare interactions, thus highlighting the need for a virtual training on HPV vaccine communication skills (Table 2). The project team met with members of the NorTex Clinical Advisory Board for their input on the best way to implement such training. The recommendation was for a virtual, asynchronous training video so that clinic team members could complete the training on their timeline. This was especially highlighted because of the COVID-19 pandemic and another wave of the virus.

**Table 2.** HPV CHAT Implementation Using the Plan-Do-Study-Act (PDSA) Framework (Cycle 1).

Cycle 1: September 1, 2021-August 31, 2022			
*Needs:* • Virtual training on HPV vaccine communication skills *Clinics:* • Safety-Net Hospital clinics, Practice-Based Research clinics, Health Department Immunization clinics			
Plan	Do	Study	Act
*Goals* • By August 2022, the program team will have trained 8 clinics and immunization outreach staff on HPV vaccine communication skills. • By August 2022, 50% of clinic trainees will report feeling confident in their ability to talk with patients about HPV vaccination and 70% of trainees will be knowledgeable about the HPV vaccine guidelines.	*Training material development* • Created training video and corresponding educational materials. • Received feedback on training content and modality of offering from the NorTex Clinical Advisory Board *Implementation* • Piloted training in practice-based research clinics • Tracked participation and completion of training • Sent recruitment emails to program/clinic managers • Provided individual and clinic incentives to increase participation (water bottles to individual participants and clinic-wide lunches based on clinic-level participation) • Created monthly evaluation reports on HPV vaccination rates	*Outcomes* • 187 clinic personnel completed the training and surveys ○ Overall response rate: 58.3% ○ RR: 51.5% (safety-net clinics) ○ RR: 50.8% (practice-based research clinics) ○ RR: 100% (county immunization clinics). • Knowledge metrics (% correct) ○ Age for first dose (84%) ○ 2-dose schedule (88%) ○ 27-45 years old (89%) ○ Cancers that can be prevented (48%) • Self-efficacy (% agree/strongly agree; very/extremely confident) ○ Answering parents’ questions (83%) ○ Providing information to parents (84%) ○ Promoting pro-immunization culture (82%) ○ Addressing parent concern about safety (69%) • Providing a strong recommendation (75%)	*Successes* • Asynchronous training video allowed providers to complete training on their own time • Recruitment emails and incentives motivated participation • Monthly reports helped in evaluating HPV vaccination rates *Barriers* • Providers’ buy-in and high turnover rate

Abbreviation: RR, response rate.

The *Do* phase involved the development of a 20-min asynchronous training video that covered HPV vaccination guidelines, evidence-based strategies for HPV recommendation, and evidence-based responses to HPV vaccine-related questions. Training was first piloted in 2 practice-based research clinics. Recruitment emails were sent to the program and clinic managers. We tracked participation rates among providers, and incentives were provided to increase participation. Monthly audit-and-feedback data reports were generated for each clinic to monitor HPV vaccination rates. To gauge the impact of HPV CHAT training, pre- and post-evaluation surveys were administered alongside the training sessions. Educational materials were also provided for patients.

For the *Study* phase, 187 participants completed the training and surveys. Participation rates were 51.5% (safety-net hospital clinics), 50.8% (practice-based research clinics), and 100% (county immunization clinics).^25^ Overall, there was a slow initial response to participation. For all training participants, more than 80% had knowledge about the age for first HPV dose, 2-dose schedule, and HPV vaccine recommendation for 27-to-45-year-olds. However, only 48% of participants knew the 6 cancers that can be prevented by the HPV vaccine. More than 80% of participants were confident about answering questions and giving information about the HPV vaccine. About 69% reported they had confidence about addressing parent concerns about the HPV vaccine safety, and about 75% were confident about providing a strong HPV vaccine recommendation. Reported values are from the post-surveys.

In the *Act* phase, we achieved most of our goals: training clinic teams and evaluation of providers’ knowledge on HPV vaccine guidelines and self-efficacy on communicating about HPV vaccination. However, there was a high turnover rate in some clinics and some providers had difficulty completing the surveys. Clinic teams were receptive to an asynchronous training video as they could participate at their own pace. Upon reflection on the next cycle, we planned to initiate strategies that would increase clinic participation, including more effective recruitment strategies and monitoring pre- and post-surveys.

### Cycle 2 (September 2022-August 2023)

Results from Cycle 1 were used to inform Cycle 2 (see Table 3).

**Table 3.** HPV CHAT Implementation using the Plan-Do-Study-Act (PDSA) Framework (Cycle 2).

Cycle 2: September 1, 2022-August 31, 2023			
*Needs:* • Efficient ways to track pre-and-post surveys • Include more HPV and HPV vaccine content in training video • Tailored recruitment strategies to include under vaccinated patient populations *Clinics:* • Safety-Net Hospital clinics, Federally Qualified Health Center clinics			
Plan	Do	Study	Act
*Goals* • By August 2023, the program team will have trained an additional 6 clinics on HPV vaccine communication skills. • By August 2023, 50% of clinic trainees will report feeling confident in their ability to talk with patients about HPV vaccination and 70% of trainees will be knowledgeable about the HPV vaccine guidelines.	*Training material development* • Included the HPV and HPV vaccine 101 education component to training video • Created an educational poster (pediatric rooms) • Linked pre/post/video • Added provider focused questions related to communication and communication intention *Implementation* • Sent recruitment emails to program/clinic managers • Posted recruitment material in safety-net clinic lunchrooms • Facilitated introduction meetings with providers (safety-net clinics + FQHCs) • Provided individual and clinic incentives to increase participation (water bottles + gift cards) • Created audit and feedback reports on HPV vaccination rates (safety-net clinics, clinic level data; FQHCs, provider level data)	*Outcomes* • 131 clinic personnel completed the training and surveys ○ Overall response rate: 60.6% ○ RR: 57% (safety-net clinics) ○ RR: 85% (FQHCs) • Knowledge metrics (% correct) ○ Age for first dose (76%) ○ 2-dose schedule (78%) ○ 27-45 years old (71%) ○ Cancers that can be prevented (23%) Self-efficacy (% agree/strongly agree) ○ Answering parents’ questions (79%) ○ Providing information to parents (82%) ○ Promoting pro-immunization culture (80%) ○ Addressing parent concern about safety (70%) ○ Providing a strong recommendation (85%)	*Successes* • Asynchronous training video allowed providers to complete training on their own time • Recruitment emails, introduction meetings via leadership, additional prompts, and incentives motivated participation • Monthly reports helped in evaluating HPV vaccination rates Barriers • Leadership changes • Clinic workload

Abbreviations: FQHCs, Federal Qualified Health Centers; RR, response rate.

For the *Plan* phase, we identified the need to initiate more effective ways to track the pre- and post-evaluation surveys, increase the basic HPV and HPV vaccine content for the training video, and tailor clinic recruitment strategies to include under-vaccinated patient populations.

The *Do* phase involved incorporating feedback from the clinics, which aided the updating of training materials. We included more information about HPV and the HPV vaccine to the training video, linked the pre- and post-evaluation surveys to the video, and created an educational poster on HPV vaccination for the clinic rooms. Additionally, we included questions on communication and communication intentions in the evaluation surveys. Clinic managers helped to facilitate introductory meetings with providers to increase participation in the training. Recruitment materials were also posted in lunchrooms. Monthly audit-and-feedback data reports on HPV vaccination rates were also created for clinics.

For the *Study* phase, 131 participants completed the training and evaluation surveys. Participation varied across settings due to strength of clinic partnerships and prior familiarity of the implementation process. For safety-net clinics, 57% of clinic personnel participated, whereas 85% of the FQHC personnel participated. Overall, the participation rate was 60.6%. Across all participants, less than 80% had knowledge about the age for first HPV dose, 2-dose schedule, and HPV vaccine recommendation for 27-to-45-year-olds. Only 23% of participants knew the 6 cancers that can be prevented by the HPV vaccine. More than 80% of participants were confident about giving information about the HPV vaccine and providing a strong recommendation. About 70% reported they had confidence about addressing parent concerns about the HPV vaccine safety.

At the *Act* phase, we had higher participation rates in Cycle 2 than Cycle 1, lower knowledge levels about HPV vaccine guidelines, and increased self-efficacy to engage in conversations about HPV vaccination. It is unknown what contributed specifically to lower HPV vaccination knowledge in cycle 2. We identified barriers that may have influenced our outcomes, such as leadership changes, clinic workload, and data reporting of HPV vaccination rates from the participating clinics.

## Discussion

Using the PDSA principles, this paper describes the process improvements observed between 2 implementation cycles of HPV CHAT. Given the emphasis placed on provider recommendations and clinic-based interventions to improve HPV vaccination, QI initiatives have been a useful approach to bring evidence to practice. Nevertheless, QI initiatives should be evaluated during the process to examine challenges and lessons learned for future work. This paper described the key lessons learned from a QI initiative in North Texas to improve clinic team HPV vaccine recommendations. While the study successfully adapted to rising barriers, the team reflected on the importance of seeking feedback from advisory boards, securing buy-in from leadership and/or influential providers, and being nimble in data acquisition efforts.

Primary care practice-based research networks, such as NorTex, are important for conducting research that is relevant to real world practice. NorTex is a network of primary care clinics/clinicians and researchers who have agreed to participate in research and collaborate with each other. NorTex is governed by both a clinical advisory board and a community advisory board. The clinical advisory board members play a critical role in the implementation of QI initiatives and research studies by identifying barriers and facilitators to conducting projects in a clinic. The community advisory board reviews projects to make sure they are acceptable to the community and are culturally appropriate. Presenting the HPV CHAT project to the NorTex boards provided an opportunity for feedback about how to best design and implement the initiative, what content would be relevant to clinicians and clinic staff, education that may be of interest to patients, and potential barriers that may arise. As such, their input helped inform the design of the training (format, length, information to be included) and strategies for implementation. The level of participation captured by the response rates across the 2 implementation cycles was an indicator of the feasibility and reach of HPV CHAT. We worked with program and clinic managers to identify strategies to increase recruitment and participation. Interventions that are tailored to the specific needs of health care settings may motivate higher engagement, particularly when aligned with routine clinical activities.

In an effort to provide flexibility to participants in this QI initiative, we initially had separate instruments for the collection of pre-post data for the asynchronous training. We realized that by separating the two, we had loss to follow-up for the post-test to fully capture the outcomes from the training. Loss to follow-up data was handled by using responses from participants who completed pre- and post-surveys. The loss to follow-up may have overestimated the effect of the training as those who were paying closer attention and/or more motivated to participate continued with the post-test and potentially had higher knowledge scores and more favorable attitudes. Our team readjusted in Year 2 to streamline the data collection process based on these findings. Although not described in this paper, the quality improvement team also had to be nimble in how outcome data related to HPV vaccination were collected through different clinical systems and without burdening clinic teams. We used 3 separate processes to collect all the same types of HPV vaccination data^36^ including (1) working directly with embedded epidemiology teams to pull electronic health record data, (2) working with data managers in the clinic to pull aggregate data in Excel, and (3) working with information technology representatives to create data dashboards. Cross-sector QI initiatives need to be flexible with data collection to evaluate the initiative in a manner that maintains rigor of the evaluation and reduces burden on clinical teams.

### Strengths and Limitations

Strengths of this study include the use of the PDSA framework to inform the reporting of our lessons learned paper, thus providing a systematic reflection on implementation activities. The diversity of the participating clinics was a good opportunity to evaluate the HPV CHAT training in different health care settings, which can be used to inform future HPV vaccination interventions. Additionally, the diverse implementation strategies such as asynchronous training, in-person interactions, incentives, monthly evaluation reports, and feedback mechanisms were instrumental to increasing participation rates among providers.

Our study also has some limitations. First, we are unable to monitor the application of the content provided by the HPV CHAT training among the providers. Providers may be motivated to engage during the program period; however, consistent use of evidence-based communication strategies is necessary to sustain HPV vaccination rates. Ongoing tools or support, such as program refresher materials or patient flyers may help sustain program outcomes. Incorporating principles of quality improvement initiatives into clinic-based protocols may help sustain the positive impacts of QI initiatives, such as HPV CHAT. Second, our results highlight short-term impacts. Therefore, measuring the effectiveness of the program with longer-term follow-up periods are needed.

Finally, the context in which the QI project was implemented must be considered. The first cycle of implementation occurred during a subsequent wave of the COVID-19 pandemic resulting in clinic team burnout from vaccination related topics. Moreover, clinic teams had competing priorities with COVID-19 related pandemic response and emerging vaccine hesitancy, which may have contributed to the implementation of this QI initiative.

### Implications for Practice and Policy

Lessons learned from this study can inform future HPV interventions at health care settings. Our QI initiative also emphasizes the need for program planners and health care professionals to consider contextual factors including organization needs, and collaborative partnerships in the development and implementation of health care interventions. Specific implementation strategies tailored to health care settings can improve the adoption and success of health care interventions or programs. The virtual training format provided convenient accessibility for all providers, while delivering consistent information to support conversations around HPV vaccination. This affirms the need to implement evidence-based programs that will promote standardized care and evaluations within health care settings. Furthermore, health care policies should prioritize the effective translation of clinical guidelines into routine practice, including evidence-based training for providers. By addressing clinical barriers, such as high clinic workloads, inadequate provider training, and limited resources, policies can improve the quality and efficiency of health care delivery. Additionally, our study emphasizes the need to initiate studies that will evaluate the impact of these interventions over time. Healthcare providers are instrumental to the success of healthcare interventions, highlighting the importance of further investigating individual and contextual factors (clinical barriers and facilitators) that influence sustained engagement.

## Conclusions

Using the iterative PDSA framework, we explored the implementation strategies initiated during each cycle of HPV CHAT. The support from health care system leaders and the asynchronous format of the virtual training were instrumental in the implementation of HPV CHAT. Despite challenges encountered, in-person interactions, follow-up emails, and incentives were effective in increasing provider recruitment and engagement. Additionally, monthly evaluation reports highlighted the HPV vaccine administration trends during the initiative. This study underscores the need to tailor dissemination and implementation strategies to effectively integrate interventions within health care settings.
